# The Effect of Local Therapy on M1c Prostate Cancer Patients: A Systematic Review and Meta-Analysis

**DOI:** 10.3389/fsurg.2021.648676

**Published:** 2021-04-22

**Authors:** Zhenghao Wang, DeHong Cao, Wuran Wei

**Affiliations:** Department of Urology, Institute of Urology, West China Hospital, Sichuan University, Chengdu, China

**Keywords:** prostate cancer, metastatic, M1c, radical prostatectomy, radiation therapy

## Abstract

**Background:** A systematic review and meta-analysis was conducted to explore the effect of local treatment (LT) on overall survival (OS) and cancer-specific mortality (CSM) for patients diagnosed with M1c prostate cancer (PCa).

**Methods:** PubMed, Web of Science, Embase, EBSCO, and Cochrane library databases (updated November 2020) were searched for studies assessing the effect of LT on patients with M1c Pca. The search strategy and study selection process was managed according to the PRISMA statement.

**Results:** Four cohort respective studies were identified for satisfying the inclusion criteria. Our results indicated that LT significantly improved CSM (*HR* = 0.36, 95% *CI* = 0.22–0.60; *P* < 0.0001) and OS (*HR* = 0.42, 95% *CI* = 0.24–0.77; *P* = 0.004). Subgroup analysis showed that radical prostatectomy (RP) and radiation therapy (RT) including brachytherapy (BT), conformal radiation therapy (CRT), and intensity modulated radiation (IMRT) had a significant benefit on cutting down the CSM of M1c PCa patients (*HR* = 0.27, 95% *CI* = 0.13–0.56; *P* = 0.0005 and *HR* = 0.42, 95% *CI* = 0.20–0.89; *P* = 0.02). In addition, RP had improved the OS for patients (*HR* = 0.33, 95% *CI* = 0.15–0.73; *P* = 0.008). There was no difference of OS in patients that underwent RT (*HR* = 0.58, 95% *CI* = 0.24–1.40; *P* = 0.23). No significant heterogeneity was among the results, indicating consistency in the study.

**Conclusions:** Present meta-analysis indicates that LT for M1c PCa correlated with decreased CSM and enhanced OS. The survival benefit of RP was successfully confirmed and the advantage of RT seemed to be associated with the tumor burden and method of RT.

## Introduction

Prostate cancer (PCa) is one of the most frequent malignancies encountered in males worldwide with around 127,106 patients diagnosed annually (1, 2). Although PCa commonly follows an indolent course with an estimated 98.9% 5-year survival, it still ranks as the second cause of mortality worldwide (3). Surgery or radiation therapy (RT) is the mainstay for the treatment of localized PCa with a high efficacy (4). Despite this, 20% of patients suffer from lymph node metastasis while about 4% have distant metastasis at diagnosis which is often associated with higher morbidity (2). Current European Association of Urology (EAU) guidelines recommend the use of androgen deprivation therapy (ADT) with or without chemotherapy for metastatic prostate cancers (mPCa) (5).

The “premetastatic niche” theory put forward by Kaplan et al. in 2006 has garnered attention toward the treatment of primary tumors. It was suggested that the primary tumor could act as the main source of metastasis through circulating tumor cells playing an important role in tumor progression (6). The benefits of local treatment (LT) for primary tumor in patients with metastatic tumors have successfully been confirmed ovarian, breast, and renal cancer (7–9). In these studies, the cytoreductive treatment significantly prolonged survival and reduced mortality by reducing the overall tumor burden and interrupting the re-seeding of the primary tumor (10). The progress in laparoscope surgery and radiation techniques has made the radical prostatectomy (RP) and radiotherapy safer and more efficient, and many urologists have successfully explored the way for the LT of mPCa (11).

In recent years, some original articles and meta-analyses have proven that LT potentially prolongs the survival in patients with mPCa (12). However, in HORRAD trial, Boevé et al. found that adding radiotherapy did not prolong the survival to patients with bone metastatic PCa (13). And in STAMPEDE trial, Parker et al. reported that the radiotherapy did not improve overall survival (OS) for unselected mPCa patients (14). Furthermore, these reports have generally ignored to perform the detailed subgroup analyses for patients with M1c prostate cancer. M1c stage PCa is the terminal stage with a poor prognosis (15) and the present evidence of LT for M1c PCa is controversial and insufficient. Therefore, this systematic review and a meta-analysis was performed to find out the effect of LT on OS and cancer-specific mortality (CSM) for patients diagnosed with M1c PCa.

## Materials and Methods

This systematic review and meta-analysis followed the guidelines of the Preferred Reporting Items for Systematic Reviews and Meta-analysis (PRISMA) statement and the Cochrane Handbook for Systematic Reviews of Interventions (16). Ethical approval and patient consent were not required because all analyses were based on previously published studies.

### Literature Search and Selection Criteria

We systematically searched several databases including PubMed, EMbase, Web of science, EBSCO, and the Cochrane Library from inception to November 2020. The research strategy consisted of the following keywords: “prostate cancer,” “metastatic,” “M1c,” “radical prostatectomy,” “radiation therapy,” and “local therapy.” The reference lists of retrieved studies and relevant reviews were hand-searched, and the process mentioned above was repeatedly performed to ensure the inclusion of all eligible studies. Inclusion criteria were as follows: (1) case-control studies, big cohorts or randomized control trials, (2) data of patient with M1c PCa (American Joint Committee on Cancer, AJCC), (3) full text only, and studies with all languages were included, (4) sufficient data for extraction, (5) intervention treatments are LT vs. NLT (no local treatment).

### Data Extraction and Outcome Measures

Baseline information extracted from the original studies included: first author, published year, study design, number of samples, follow-up time, end point indicator, and method details for the two groups. Data were independently extracted by two investigators and any discrepancy was resolved by consensus. The outcomes contain OS and CMS for patients with M1c prostate cancer.

### Quality Assessment of Individual Studies

All assessments were performed independently by two researchers with differences resolved by discussion to reach the third researcher. The methodological quality assessment of eligible studies was evaluating by the Newcastle–Ottawa Scale (NOS) (17). There are eight quality assessment criteria: (1) representativeness of the exposed cohort; (2) selection of the non-exposed cohort; (3) ascertainment of exposure; (4) outcome of interest does not present at start of study; (4) outcome of interest does not present at start of study; (5) control for important factor or additional factor; (6) assessment of outcome; (7) follow-up long enough for outcomes to occur; (8) adequacy of follow up of cohorts. Each quality choice could be awarded a maximum of one star except for the numbered five item which could be granted a maximum of two stars. Total quality scores ranged from 0 to 9. If the final score >6, we regarded it as high quality.

### Statistical Analysis

The hazard ratio (*HR*) and 95% confidence interval (95% *CI*) were extracted directly from the study reports. If insufficient data were available, supplementary data might be sought directly from the investigators of studies. A fixed-effect model or random effect model was used for analyses based on heterogeneity among studies. We used the Chi-square and the *I*-square tests to assess the heterogeneity among the studies. Chi-squared with a *P* < 0.10 or *I*-square >50% was considered as significant heterogeneity. Sensitivity analysis was performed for evaluating the influence of a single study on the overall estimate by omitting one study in turn or performing subgroup analysis. All statistical analyses were performed using Review Manager Software Version 5.3 (The Cochrane Collaboration, Software Update, Oxford, UK).

## Results

### Literature Search, Study Characteristics, and Quality Assessment

**Figure 1:**
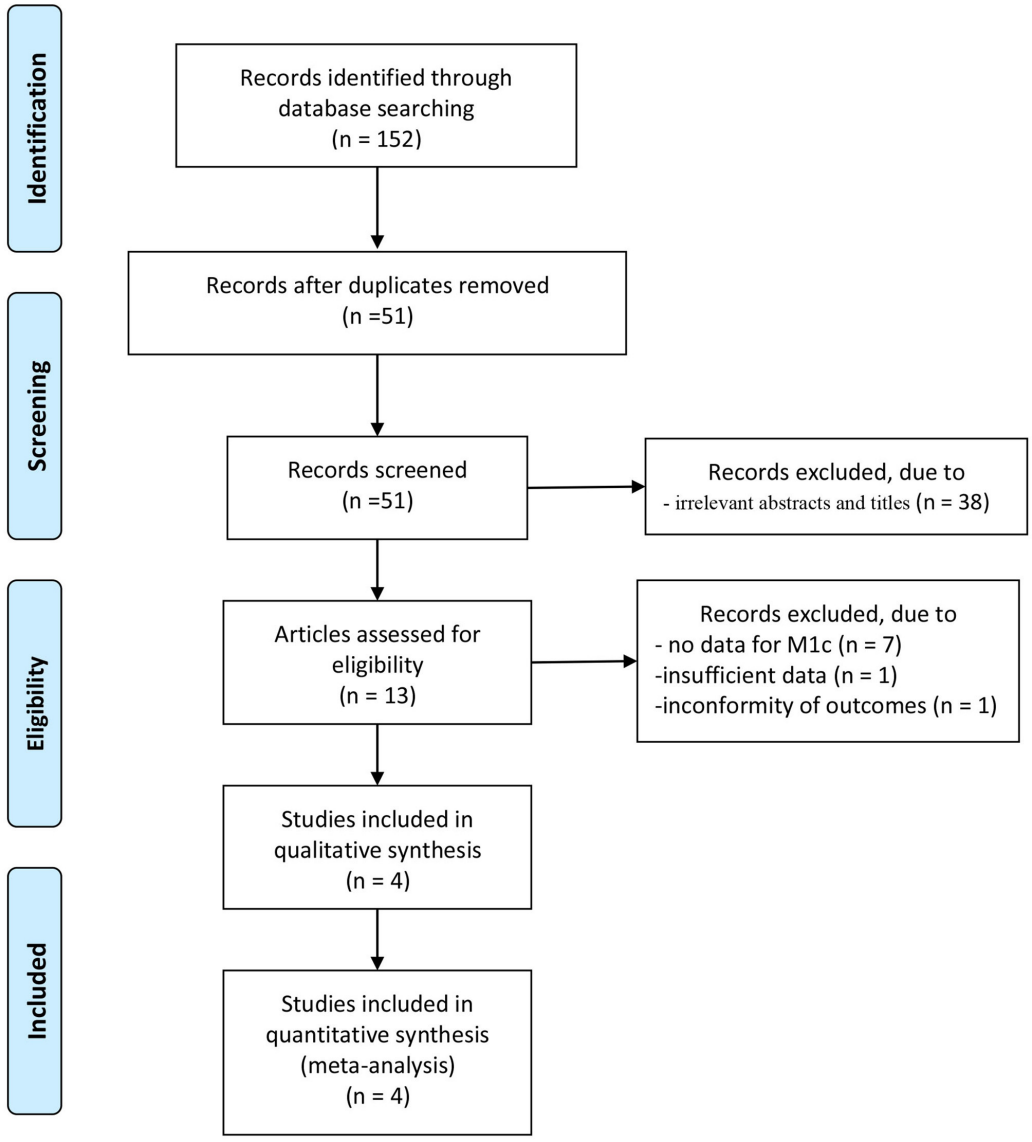
Flow diagram of study searching and selection process.

A total of 152 articles were initially identified from database searches. After the removal of duplicates, 51 articles were retained. Of these, 38 were excluded from analysis following the screening of the abstracts and titles, 7 were excluded as the data for patients with stage M1c were not counted separately, one was excluded owing to insufficient data, while another was excluded for its non-conforming outcomes. Four cohort retrospective studies with total 34,018 patients (from inception to November 2020) were identified for satisfying the inclusion criteria, and they were finally enrolled in this meta-analysis (18–21). The article selection process was performed per the PRISMA guidelines (Figure 1). Baseline characteristics of the four included studies are shown in Table 1. These studies were published between 2014 and 2018.

**Table 1:** Characteristics of included studies.

**Study**	**Design**	**Source of HR**	**Follow-up**	**Sample**	**LT group intervention**	**NLT group intervention**	**End point**	**NOS**
			**(months)**				**(MA)**	
Antwi and Everson (18)	CR	Reported	80 maximum	7,858	RP or BT	NLT	CSM	7
Culp et al. (19)	CR	Reported	27 median	8,185	RP or BT	NLT	OS	6
Satkunasivam et al. (20)	CR	Reported	20 median	4,069	RP or CRT/IMRT	NLT	OS/CSM	7
Pompe et al. (21)	CR	Reported	31.5 median	13,906	RP or BT	NLT	CSM	7

*CR, Cohort retrospective; LT, Local therapy; NLT, No local therapy; IMRT, intensity modulated radiation; CRT, conformal radiation therapy; RP, radical prostatectomy; BT, brachytherapy; OS, overall survival; CSS, Cancer survival specific; MA, multivariate analysis; NOS, Newcastle–Ottawa Quality*.

Three studies take CSM as the outcome, Satkunasivam et al. (20) reported both the OS and CSM as the outcome and Culp et al. (19) only reported the OS. In all the studies, patients were subjected to RP and RT. In three of the four studies, brachytherapy (BT) was performed and one study use intensity modulated radiation (IMRT)/conformal radiation therapy (CRT) as an intervention in subgroup. Pompe et al. (21) reported the outcome of a combination of the RP and RT and other study reported the outcomes separately associated with each intervention.

A funnel plot was not conducted due to the limited number of included studies. The NOS scores of the included studies varied from 6 to 7. Three studies (18–21) were evaluated as high quality (scores 7) and 1 study (19) was evaluated as median quality (score 6).

### The Effect of LT for M1c Prostate Cancer on CSM

**Figure 2:**
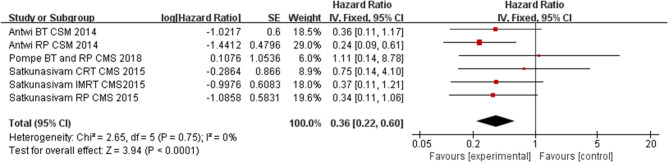
Forest plot of HR for CSM following LT.

**Figure 3:**
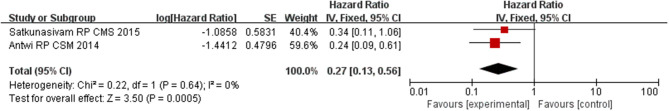
Forest plot of HR for CSM following RP.

**Figure 4:**
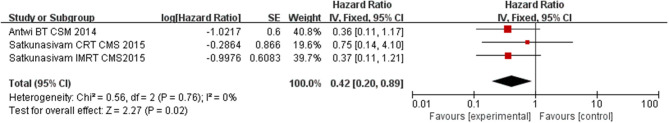
Forest plot of HR for CSM following RT.

Compared to NLT groups, the use of LT significantly improved CSM for the patients (*HR* = 0.36, 95% *CI* = 0.22–0.60; *P* < 0.0001) with insignificant heterogeneity among the studies (*I*^2^ = 0%, *P* = 0.75, Figure 2). In the subgroup analysis for each intervention, we found RP and RT (including BT, CRT, and IMRT) having a significant benefit on cutting down CSM of M1c PCa patients (*HR* = 0.27, 95% *CI* = 0.13–0.56; *P* = 0.0005 and *HR* = 0.42, 95% *CI* = 0.20–0.89; *P* = 0.02) with insignificant heterogeneity among the studies (*I*^2^ = 0%, *P* = 0.64, Figure 3; *I*^2^ = 0%, *P* = 0.76, Figure 4).

### The Effect of LT for M1c Prostate Cancer on OS

**Figure 5:**
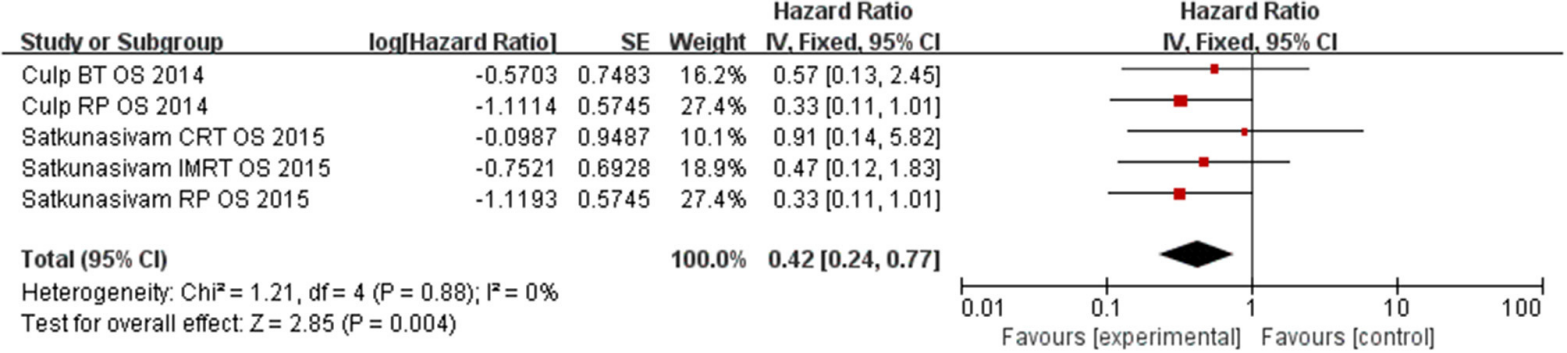
Forest plot of HR for OS following LT.

**Figure 6:**
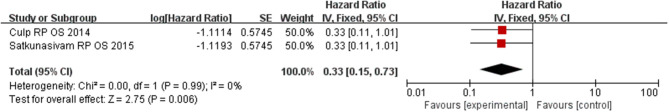
Forest plot of HR for OS following RP.

**Figure 7:**
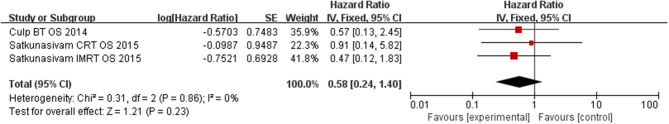
Forest plot of HR for OS following RT.

Our results find out that LT can significantly improve OS (*HR* = 0.42, 95% *CI* = 0.24–0.77; *P* = 0.004) with insignificant heterogeneity (*I*^2^ = 0%, *P* = 0.88, Figure 5). Subgroup analysis showed that RP had a benefit for OS for patients (*HR* = 0.33, 95% *CI* = 0.15–0.73; *P* = 0.008) with insignificant heterogeneity (*I*^2^ = 0%, *P* = 0.99, Figure 6). However, there was no difference was observed in the OS in patients undergoing RT (*HR* = 0.58, 95% *CI* = 0.24–1.40; *P* = 0.23) with insignificant heterogeneity (*I*^2^ = 0%, *P* = 0.86, Figure 7). No significant heterogeneity was found in the results, indicating good consistency.

## Discussion

Epidemiology studies suggest that PCa still remains the second-leading cause of cancer death in men (22). As the diagnostic techniques and treatments for mPCa continue to evolve, men with more advanced PCa are living longer (23, 24). The treatment of choice for mPCa has become complex with the increasing number of treatment options (25), as opposed to the traditional approach like ADT. Despite the better efficacy of ADT and curtail disease-related symptoms, resistance to hormone therapy ultimately develops, thus pushing cytoreductive LT to the forefront. Nevertheless, ADT is still initial management choice for metastatic Pca according to current guideline. However, for prolong survival for patients, many complements like new agents (including abiraterone, cabazitaxel, enzalutamide, and sipuleucel-T), local RT for oligo-metastatic disease, directed RT to metastases and RP for local tumor has emerged. Owing to improvement in surgical techniques, surgery as an intervention has changed from the treatment of choice in low risk to more advanced and even in high-risk PCa (26). Though present meta-analyses showed that LT significantly improved OS and CMS of patients with metastatic prostatic cancer (12, 27), however, these meta-analyses lack a subgroup analysis of M1 substage-specific which often combine with highest risk and mortality. Furthermore, Pompe et al. concluded that no survival benefit existed in M1c patients for LT, which makes the clinical effect of LT controversial (21). Therefore, the present study was conducted and to the best of our knowledge, it is the first meta-analysis to evaluate the impact on LT for M1c Pca. Despite the inherent limitations with the CR design of studies, our result indicates that LT has a significant benefit on both OS and CSM on the M1c Pca patients. Using multivariable competing risk regression analyses, Pompe et al. found a significant improvement after LT in M1a and M1b patients with a baseline PSA lower than 60 ng/ml, by 1:2 propensity score matching in SEER database. However, the patients with M1c PCa did not show a CSM benefit (18). Compared to the M1a-b, the M1c stage is often associated with a more tumor burden, but Pompe et al. only selected patients who underwent BT without an organ site-specific code. Furthermore, they reported the final CSM of RP and RT collectively rather than separately. Thus, the LT for each patient is not specific which might explain the negative results obtained in their study.

The present study indicates that RT has a positive effect on the survival of the patient specially for CSM. It's worth noting Satkunasivam et al. reported that RT may has a potential poor effect on patients with high tumor burden M1c Pca. In their study, a combination of IMRT and CRT was associated with a decreased risk of CSM (*HR*: 0.64, 95% *CI*: 0.50–0.82). However, compared to NLT, CRT was not associated with lower risk of CSM (*HR*: 0.85, 95% *CI*: 0.64–1.14). This relates to the clinical scenario where CRT may be viewed as non-definitive therapy in contemporary practice. The lower treatment doses are delivered to the tumor region compared to other definitive therapy (20). This result is also found in a recent STAMPEDE trial, they found the therapeutic effect is better to the low tumor burden and diminished in unselected M1 patients (14). Therefore, we assume that RT is an effective method for survival in patients with M1c PCa, but the efficacy is related to methods and doses of the RT as well as the tumor burden. However, present research mainly focus on the relation about bone metastatic and do not contain the separated organ metastatic (M1c) (13, 14). Furthermore, the treatment for M1c PCa patients without data from further trials should be very careful as M1c disease is mostly high burden. And the future study to design to confirm this view.

Previous research has confirmed that feasibility and the survival benefit of RP for metastatic Pca (28). Furthermore, a multivariable competing risk regression analyses tested CSM after propensity score matching in SEER database and the results showed that in comparison to RT, RP demonstrated important and statistically significant survival benefit in CSM in every stage of metastatic Pca (15). Additionally, Wang et al. reported that a relatively low level of Gleason score, M-stage or N-stage could be a better prognosis for patients with metastatic Pca undergoing RP (26). Loppenberg et al. further suggested that in choosing the right patients with metastatic Pca for surgery, less aggressive tumors and general good health should be taken into consideration which is also recommended in three ongoing prospective studies (NCT02458716, NCT01751438, and NCT02454543) (29). In our study, we found that RP has the survival benefit of decreasing CSM and improving OS for M1c Pca. Thus, we suggest that the surgical indications for metastatic Pca should not be too strict. Sooriakumaran et al. has retrospectively studied the complications for 106 patients and found RP is reasonably safe approach for men with metastatic PCa. However, Only M1a/M1b subgroups were examined in their study (30) therefore, to understand this approach, more data is required future study.

Our study had a few limitations, first, the nature of study design is a major limitation of this study, the number of studies is relatively small and the patients' characteristics like co-mobilities, tumor burden (location of metastases, number of metastases), previous treatments are lacked in original studies. Second, there was difference in tumor burden in four studies which made assessing the true efficacy of LT complicated (especially for different method of RT). Third, because of the limited data, the indication and risks of these two approaches were seldom involved. Lastly, there are some unpublished data and missing negative data in the original reports, due to which publication bias may have creeped in and skewed our conclusion. Thus, the high level prospective RCT with large sample size and a more consistent baseline for patients should be made in the future studies to offer a comprehensive and accurate data.

In conclusion, the present meta-analysis indicates that LT for M1c PCa correlated with decreased CSM and an enhanced OS. Based on our results, the survival benefit of RP was successfully confirmed, and the benefits of RT may be associated with the tumor burden and the method of RT. Besides, it is important to consider our study limitations until more high-level evidence to verify our results.

## Data Availability Statement

The original contributions presented in the study are included in the article/supplementary material, further inquiries can be directed to the corresponding author/s.

## Author Contributions

ZW contributed to the conceptualization, data curation, formal analysis, investigation, methodology, project administration, validation, and writing—original draft. WW contributed to the supervision. ZW and DC contributed to the resources, software, visualization. All authors contributed to the article and approved the submitted version.

## Conflict of Interest

The authors declare that the research was conducted in the absence of any commercial or financial relationships that could be construed as a potential conflict of interest.
